# Supplementary material for: Multi-scale structure and topological anomaly detection via a new network statistic: The onion decomposition
Source: Sci Rep. 2016 Aug 18;6:31708. doi: 10.1038/srep31708 (PMC4989159; doi:10.1038/srep31708)
Supplement: Supplementary Information [file srep31708-s1.pdf]

Supplementary Information for "Multi-scale structure and topological anomaly detection via a new network statistic: The onion decomposition"

Laurent Hébert-Dufresne,[1] Joshua A. Grochow,[1] and Antoine Allard[2]

[1]Santa Fe Institute, Santa Fe, NM 87501, USA

[2]Departament de Física de la Matèria Condensada, Universitat de Barcelona, Barcelona 08028, Spain

I. IMPLEMENTATION NOTES

Several strategies for computing ThisLayer suggest themselves; which strategy performs best may depend on how various data structures are implemented in a given programming language. One choice is to sort V and D in ascending order by degree before the main while loop for a cost of $O(|V|\log|V|)$; when neighbor degrees are decremented, they can be removed from these lists and re-inserted using binary search for a cost of $O(|N(v)|\log|V|)$, which sums to another $O(|E|\log|V|)$. The minimum degree of the remaining nodes is easily accessed in $O(1)$ time as the first element of the list D. Another choice is to maintain the nodes in a min-heap, keyed by their degree. Constructing this heap before the while loop takes time $O(|V|\log|V|)$, as before. Again, when neighbor degrees are decremented they must be re-inserted to the heap, for a cost of $O(|N(v)|\log|V|)$, also as before. The minimum degree is easily accessed in $O(1)$ time by peeking at the minimum element of the heap. Again, we emphasize that although the asymptotic run-times of these two methods are the same up to big-Oh (and even possibly to lower order terms), which performs better may depend on the exact implementation of these algorithms and data structures, either by the user or in a language-provided library.

It may also be advantageous to remove nodes in a single sweep over V each time, rather than to first compute ThisLayer and then remove them all at once. For this, we suggest that the following notion of "effective degree" of a node in shell k is useful for avoiding errors. The effective degree of a node in shell k is defined as its number of links leading to nodes in shells $k' \geq k$. At each step of the algorithm, a layer is peeled by removing all nodes of effective degree equal to the minimal effective degree in the network and diminish the effective degree of their neighbours only if it is higher than their own.

II. ADDITIONAL ANALYSIS OF TOY MODELS

Supplementary Figure 1 presents two of the toy networks studied in the main text. As it was mentioned, the perfect tree has an exponentially decaying spectrum while that of the lattice is linear (both in its increasing and decreasing regimes). We now demonstrate these conclusions analytically.

In the case of the **Cayley tree**, the network is created by starting with a node (constituting "ring 0") connected to z neighbours (ring 1) and repeating a branching factor $z-1$ around the leaves for every additional ring $r > 1$. This results in a tree with $L(r) = z(z-1)^{r-1}$ nodes in every ring $r > 0$ and with a total of

$$N(r_{\max}) = 1 + \sum_{r=1}^{r_{\max}} L(r) = \frac{z(z-1)^{r_{\max}} - 2}{z-2} \tag{1}$$

nodes. The OD peels the network with the inverse procedure: First removing the $z(z-1)^{r_{\max}-1}$ leaves in the final ring for layer 1, then the $z(z-1)^{r_{\max}-2}$ nodes in ring $r_{\max}-1$ for layer 2, and so on until the final node. Using the equivalence $\ell \equiv r_{\max} - (r-1)$ between rings (r) in the network creation and layers (ℓ) in the OD, we can write the following ratio between nodes contained in subsequent layers ℓ and $\ell - 1$ with $1 < \ell \leq r_{\max}$:

$$\frac{L(r_{\max} - \ell + 1)}{L(r_{\max} - \ell + 2)} = \frac{z(z-1)^{r_{\max}-\ell}}{z(z-1)^{r_{\max}-\ell+1}} = \frac{1}{z-1} \ . \tag{2}$$

This constant decay as ℓ increases results in an exponential spectrum until the final layer, $\ell_{\max} = r_{\max} + 1$, whose density is $1/z$ times the density of layer $\ell_{\max} - 1$. This spectrum is confirmed on Supp. Fig. 1. In the rewired network, all $L(r_{\max})$ nodes of degree 1 are given the same role and all $N(r_{\max} - 1)$ nodes of degree 3 are given the same role. By removing the strict constraints of the perfect tree, chances are that this rewiring process will now produce loops. Those loops force the appearance of 2− or 3−cores, thus removing nodes from the lower core and changing the purely exponential distribution of the perfect tree.

In the case of the square $L \times L$ **lattice**, the OD peels the network from the corners inward such that nodes located on a same diagonal belong to the same layer (see Supp. Fig. 1). Consequently, the onion spectrum has the shape of a triangle with each

layer having 4 more/less nodes than the previous one. More precisely, the number of nodes in each layer ℓ is given by

$$T(\ell) = \begin{cases} 4\ell & \text{for } 1 \geq \ell \geq \lfloor L/2 \rfloor \\ 4(L-\ell) & \text{for } \lfloor L/2 \rfloor < \ell < \ell_{\max} \end{cases} \tag{3}$$

$$T(\ell_{\max}) = \begin{cases} 4 & L \text{ even} \\ 1 & L \text{ odd} \end{cases}, \tag{4}$$

where $\ell_{\max} = 2\lfloor (L+1)/2 \rfloor - 1$ the total number of layers. This is confirmed on Supp. Fig. 1 for a 500×500 lattice. Also shown on Supp. Fig. 1, we see that rewiring links creates new cores and changes the unique shape of the OD: The rewired network is much faster to decompose since the linear behavior of the OD is lost.

Supplementary Figure 2 presents a different toy model not discussed in the main text: The **Ravasz-Barabási hierarchical network** model [1], which allows us to investigate the impact of self-similar hierarchy on the OD. This hierarchical network is initiated with a fully connected clique of arbitrary size s (in this case a 5-clique) in which a central node is chosen. The network is then created by replicating the current network $s-1$ times and connecting all "leaves" (which, in the $n-th$ iteration corresponds to the non-central node of the cliques that were created at the $(n-1)$-th iteration) to the central node of the original clique. An illustration with $s = 5$ and two iterations of the multiplicative process is given in Supp. Fig. 2.

In some ways, the Cayley tree is also hierarchical in the sense that the role of a node is perfectly defined by the layer in which it is found. In the case of the Ravasz-Barabási hierarchical network, nodes also have a well-defined structural role but their distance to the central node is no longer a good indicator of that role. This hierarchical network is self-similar in the sense that a base unit is repeated to obtain the full network, which is very different from simple branching. This implies that nodes of lower centrality are found everywhere in the network, including in the neighborhood of very central nodes. Hence, the network breaks down very quickly under the OD as we change the degrees of many nodes at every pass. Moreover, the self-similarity of the network appears reflected in the similarity between the internal onion spectrum of the different cores.

As with the previous toy models, the OD of a Ravasz-Barabási produced by repeating the initial motifs n times can be calculated analytically. However, there is not much value to the calculation other than realizing how the calculation of layer density at a given coreness is re-used to calculate the density of layers in the next coreness value, again reflecting the self-similar structure of the graph. In the rewired network, we lose both the fast break down of the network and the self-similarity between cores.

Supplementary Figure 3 presents the spectrum of a realization of the **stochastic block model** as a final toy network. In this realization, we used four groups of different densities to illustrate how those 4 different subgraphs are captured by the OD as 4 different cores. The layer at which those cores are found inform us on the density of the groups, and their density reflect their sizes. Of course, if two of these 4 groups had the same density, they would be merged in one core containing the nodes of both groups.

III. ADDITIONAL REAL COMPLEX NETWORKS

We now present the onion spectra of a few more real-world complex networks, most of them had their coreness (but not layers) distribution briefly studied in Ref. [2]. First, diverse **social networks** that were not shown in the main text are presented in Supp. Fig. 4. These are networks of co-authorship on a scientific pre-print archive (arXiv), "friendships" between users of a news aggregator and message board website (Digg), connections on a peer-to-peer file sharing network (Gnutella), and an old subset of Facebook from the University of Michigan.

In all cases, we see obvious signatures of clustering between active users in the sub-exponential density decay of central cores. However, we here want to focus on the positive degree correlations (assortativity). In the main text, we hinted toward the fact that assortativity tends to raise the expected number of cores in a network by joining high-degree nodes together. This is confirmed here in all cases but Gnutella (see Supp. Table I). Gnutella is an interesting case because of the very different behaviour that can be observed in the lower and higher cores. In fact, the degree distribution of the network is bimodal (not shown); already hinting at very different behaviour between central and peripheral nodes. The lower cores show signs of disassortativity in the varying decay rate between the layer of a given core (compared to the very tree-like decay of the rewired Gnutella). As seen with the Myspace network (see see Supp. Fig. 5).), this can be explained through negative degree correlations: A significant number of nodes removed in a layer of the first core are connected with hubs part of higher cores and do not contribute to the density of the next layer. Contrariwise, the higher cores of the Gnutella network show clear sign of clustering which is correlated with assortativity. In fact, most hubs are connected to each other and the 7-core is an almost fully connected clique of hubs. Those different behaviour between central and periphery nodes is reflected in the OD, but of course not captured by simply looking at a degree-degree correlation coefficient.

In all other networks, comparing the number of cores found in a real network to the number found in a rewired version appears to be a robust signature of potential degree-degree correlations. Comparing the myspace online social network spectrum with a

rewired scheme preserving those correlations confirms this conclusion (see Supp. Fig. 5). The spectrum of the myspace network also confirms that diminishing decay rate within a single core are a signature of disassortativity as suggested in the main text using the structure of a web domain.

We also use another co-authorship network, here as extracted on MathSciNet [3], to identify subgraphs of authors similar to the one highlighted in the main text using the cond-mat arXiv. Results are shown in Supp. Fig. 6. We also illustrate a similar but smaller subgraph on the arXiv network in Supp. Fig. 7.

Second, we present a few snapshots of the **Internet structure** in Supp. Fig. 8. All networks reflect the Internet structure at the level of autonomous systems. However, they use data from different time periods (all from `routeviews.org`). Similarly to the Word-Wide-Web studied in the main text, these technological networks provide great examples of negative degree-degree correlations as shown in Supp. Table I. Perhaps more importantly, they provide a great example of how the OD could be used as a method to characterize the nature of networks. In all cases, the overall patterns observed on the OD appear very robust through time: An overall tree-like structure with a very clear core of central nodes whose organisations clash with the global structure. While it was known that the k-core decomposition provided a good model for the growth of the Internet structure [4], the OD provides the first evidence for how the structure of central nodes differ from the rest. Looking for these robust patterns could guide future efforts in network characterization. Supplementary Figure 9 provides another example of this robustness by comparing the onion spectrum of the American power grid discussed in the main text to that of a Polish power grid.

Third, we revisit **World-Wide-Web domains**: `notredame.edu` and `stanford.edu` in Supp. Fig. 10. Again, as in our discussion of `stanford.edu` in the main text we find very unexpected subgraphs: e.g. long chains of nodes with the same structural role in lower cores and very dense cores in higher cores (e.g. around layer 600 of `notredame.edu`). In between these two behaviours, we find a mixture of communities coupled to a long chain of central webpages, as shown in Supp. Fig. 11.

Supplementary Table I. Comparing degree-degree correlation coefficient (r) to the ratio between the number of cores over the expected number of cores in the rewired network ($c_{\max}/\langle c_{\max}\rangle_{\text{rewired}}$). Based on our previous results, we expect the sign of r to be correlated with whether $c_{\max}/\langle c_{\max}\rangle_{\text{rewired}}$ is greater or smaller than one. Only Gnutella does not follow our intuition (for reasons covered in the text).

Network	$c_{\max}/\langle c_{\max}\rangle_{\text{rewired}}$	r
arXiv	1.895	0.147
Digg	1.000	0.004
MathSci	3.833	0.123
Gnutella	1.167	−0.103
Facebook	1.349	0.115
Myspace	0.220	−0.112
American power grid	2.5	0.003
Polish power grid	2.00	0.050
Pennsylvania roads	1.333	0.123
`notredame.edu`	0.621	−0.053
`stanford.edu`	0.300	−0.112
Internet (2005)	0.4237	−0.198
Internet (1997-2000)	0.400	−0.170
Internet (2004-2007)	0.660	−0.195
Internet (2001)	0.338	−0.164

IV. THE ONION NETWORK ENSEMBLE (ONE)

In the main text, we showed how the ONE could be useful to model a few selected networks. It is important to note that we verified the rules of construction of the ONE on all considered networks. We thus created randomized networks respecting a given onion spectrum and degree distribution, then re-ran the OD on the obtained networks to verify that the spectrum was indeed conserved by our rewiring procedure. Two of these tests, on two different non-trivial spectrum, are presented in Supp. Fig. 12.

The information required to create the ONE is the joint degree-layer distribution (which scales as the maximum degree times the number of layers), a list describing to which k-shell or coreness nodes in a given layer belong (scaling as the number of layers) and the layer-layer link correlation matrix (scaling as the square of the number of layers). The joint degree-layer distribution

being the biggest contribution to the weight of the model, the ONE can be said to scale as $k_{\max}\ell_{\max}$. On Supp. Fig. 13 we show that the number of layers consistently scales with the square root of the maximum degree $k_{\max}$. Consequently, we can say that the information needed for the **ONE roughly scales as $k_{\max}^{3/2}$**.

In Fig. 14 we show the full distribution of path lengths in real networks (when small enough to compute it), and compare it to their rewired CM, CCM and HRN versions and their respective ONEs. In all cases, networks sampled from the ONE better control for the distribution of shortest paths. In two cases, both power grids, the gain is very small since the rewiring destroys the very strict *correlated* tree structure. In other cases, as the most recent snapshot of the Internet structure, the ONE proves to be an incredibly accurate model.

[1] E. Ravasz and A.-L. Barabási, "Hierarchical organization in complex networks," Phys. Rev. E **67**, 026112 (2003).
[2] L. Hébert-Dufresne, A. Allard, J.-G. Young, and L. J. Dubé, "Percolation on random networks with arbitrary k-core structure," Phys Rev E **88** (2013).
[3] Gergely Palla, I.J. Farkas, P. Pollner, I. Derényi, and Tamás Vicsek, "Fundamental statistical features and self-similar properties of tagged networks." New Journal of Physics **10**, 123026 (2008).
[4] S. Carmi, S. Havlin, S. Kirkpatrick, Y. Shavitt, and E. Shir, "A model of internet topology using k-shell decomposition," Proc. Natl. Acad. Sci. U.S.A. **104**, 11150–11154 (2007).
[5] J. Leskovec, J. Kleinberg, and C. Faloutsos, "Graphs over time: Densification laws, shrinking diameters and possible explanations," ACM SIGKDD International Conference on Knowledge Discovery and Data Mining (2005).
[6] Laurent Hébert-Dufresne, Antoine Allard, Vincent Marceau, Pierre-André Noël, and Louis Dubé, "Structural Preferential Attachment: Network Organization beyond the Link," Phys. Rev. Lett. **107**, 1–5 (2011).

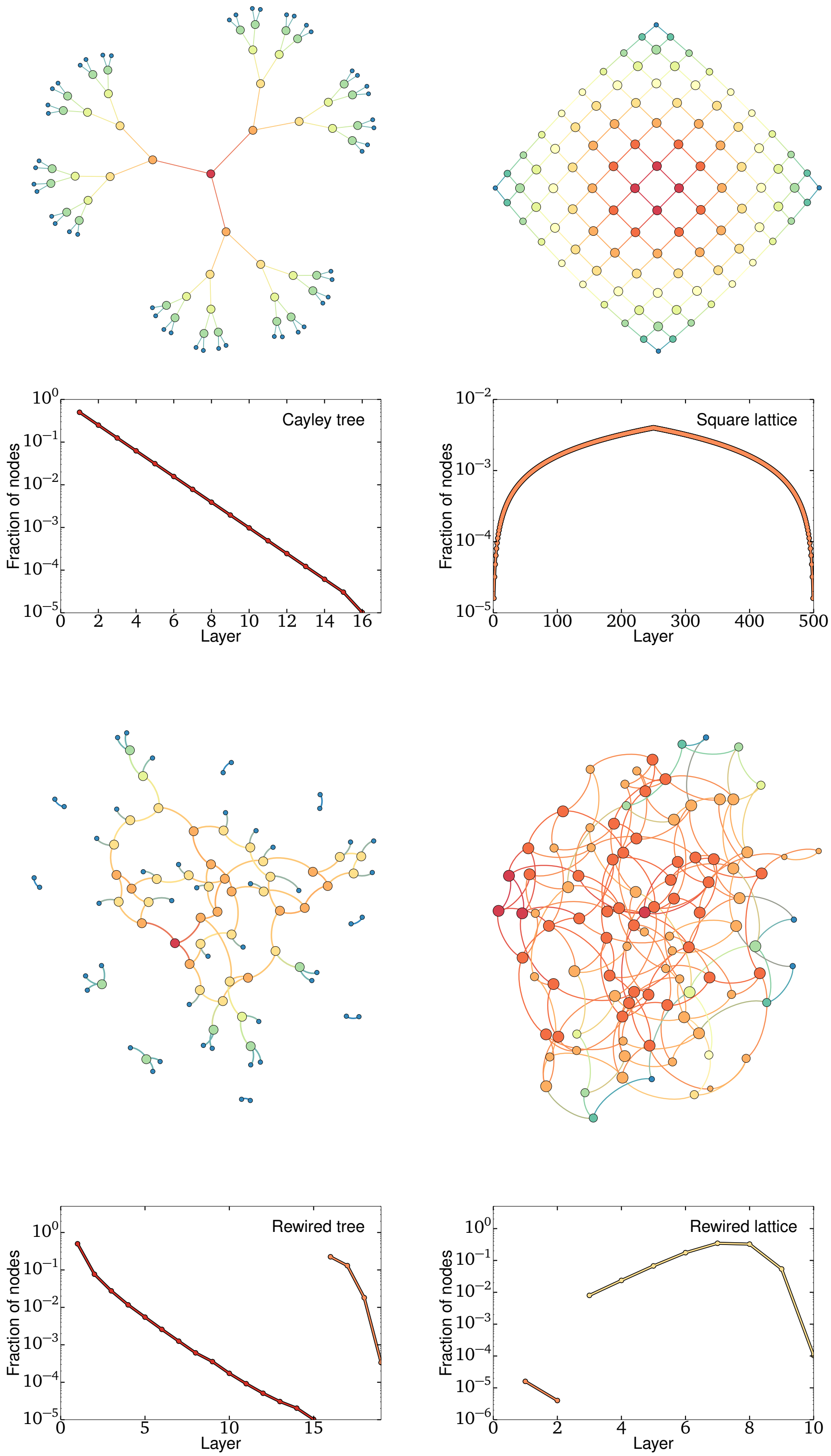

Supplementary Figure 1. **Comparing the onion spectra of toy models and their rewired version (part 1).** Illustrations of two toy networks (straight links) and their rewired versions (curved links) along with their onion spectra: (left column) Cayley tree and (right column) square lattice.

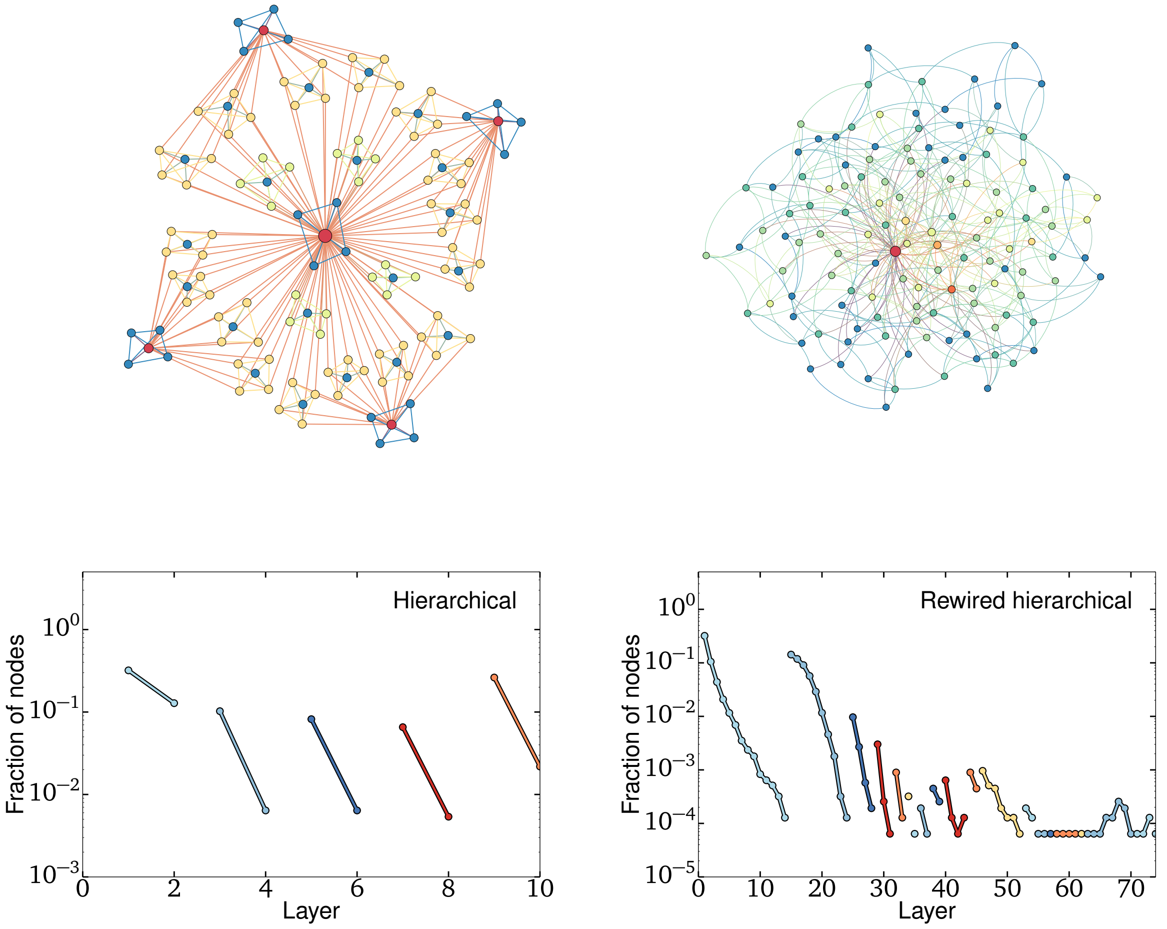

Supplementary Figure 2. **Comparing the onion spectra of toy models and their rewired version (part 2).** Illustration of the Ravasz-Barabási hierarchical network model initiated with a 5-clique and iterating the multiplicative process twice. Its rewired version is again shown with curved links. The corresponding spectra (for larger realization of those network) are shown under the illustrations.

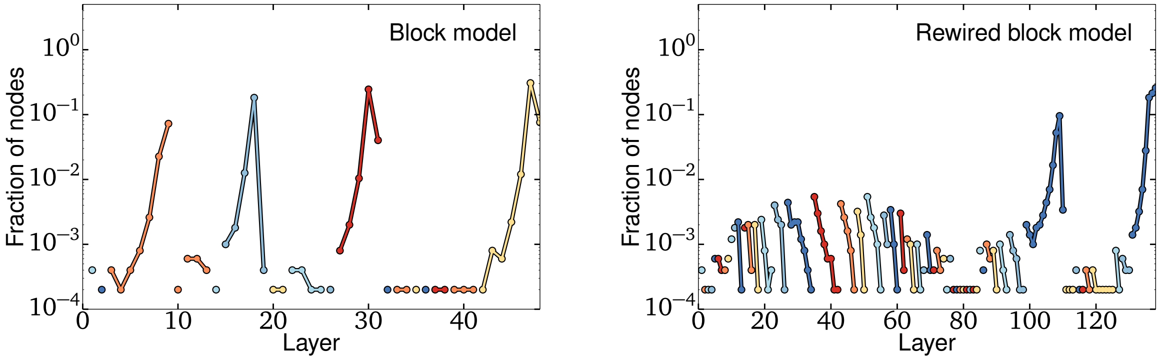

Supplementary Figure 3. **Comparing the onion spectra of toy models and their rewired version (part 3).** (left) Spectrum of a stochastic block model with 4 groups of different densities and sizes. (right) Spectrum of the rewired version.

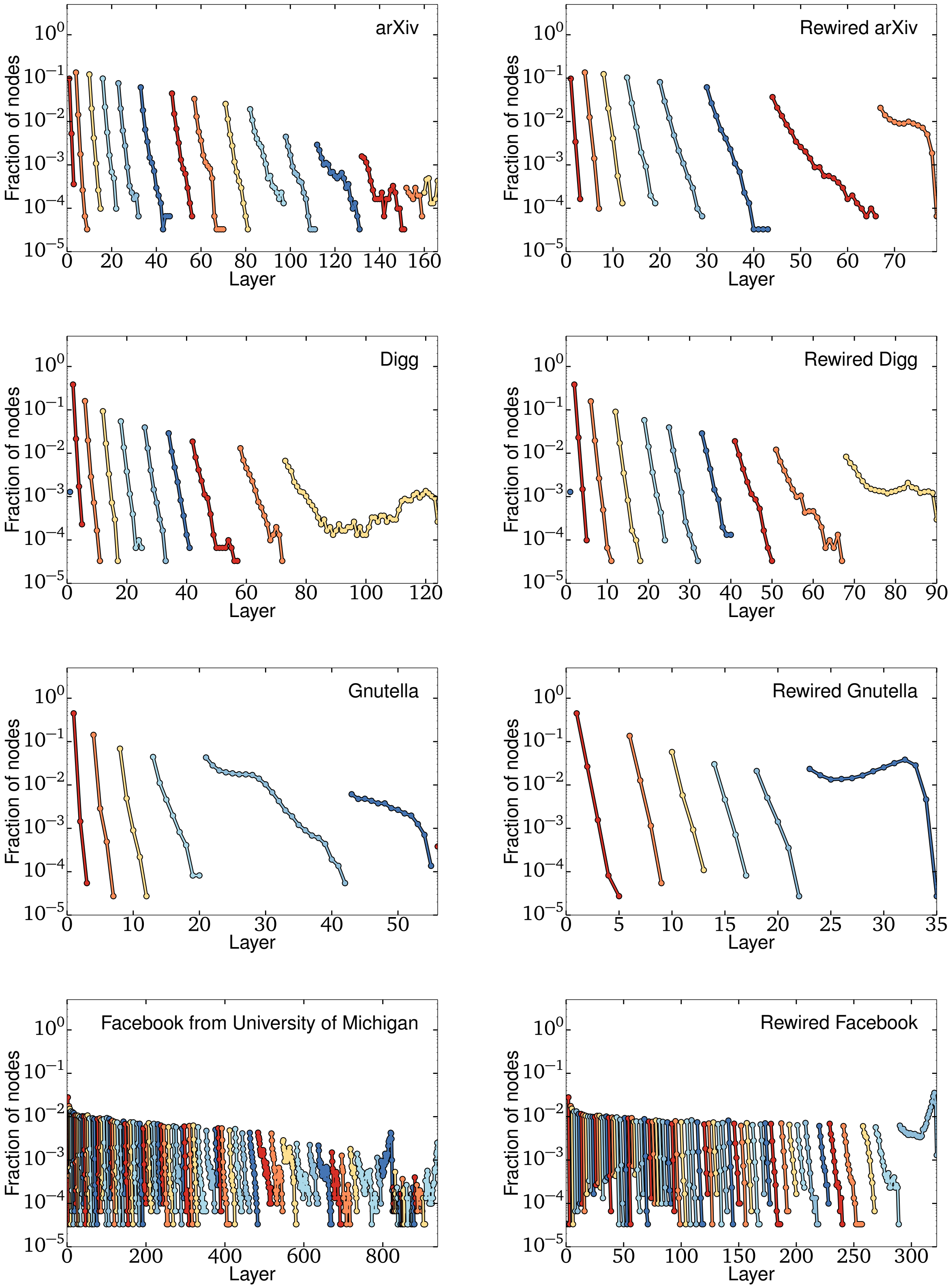

Supplementary Figure 4. **Comparing the onion spectra of social networks (left) and their rewired version (right).**

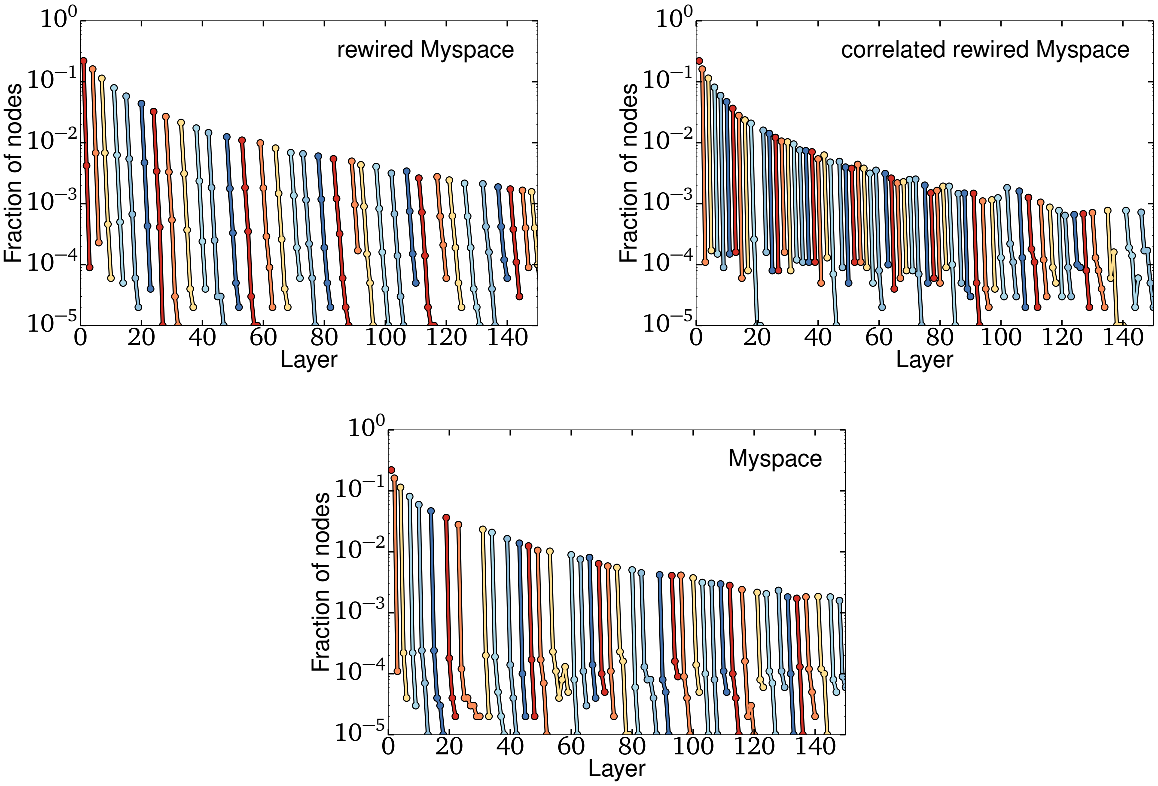

Supplementary Figure 5. **The onion spectra of the Myspace online network and of two randomized versions.** Randomizing a network for comparison can highlight some of its properties. In this case, the total number of cores and the density of their secondary layers in the Myspace network (bottom) are a sign of its negative degree correlations. This is confirmed by comparing the spectrum of Myspace to that of randomized version which removes (top left) or preserves (top right) the degree correlations. These plots only show the spectra up to the 150th layer to highlight the initial behaviour.

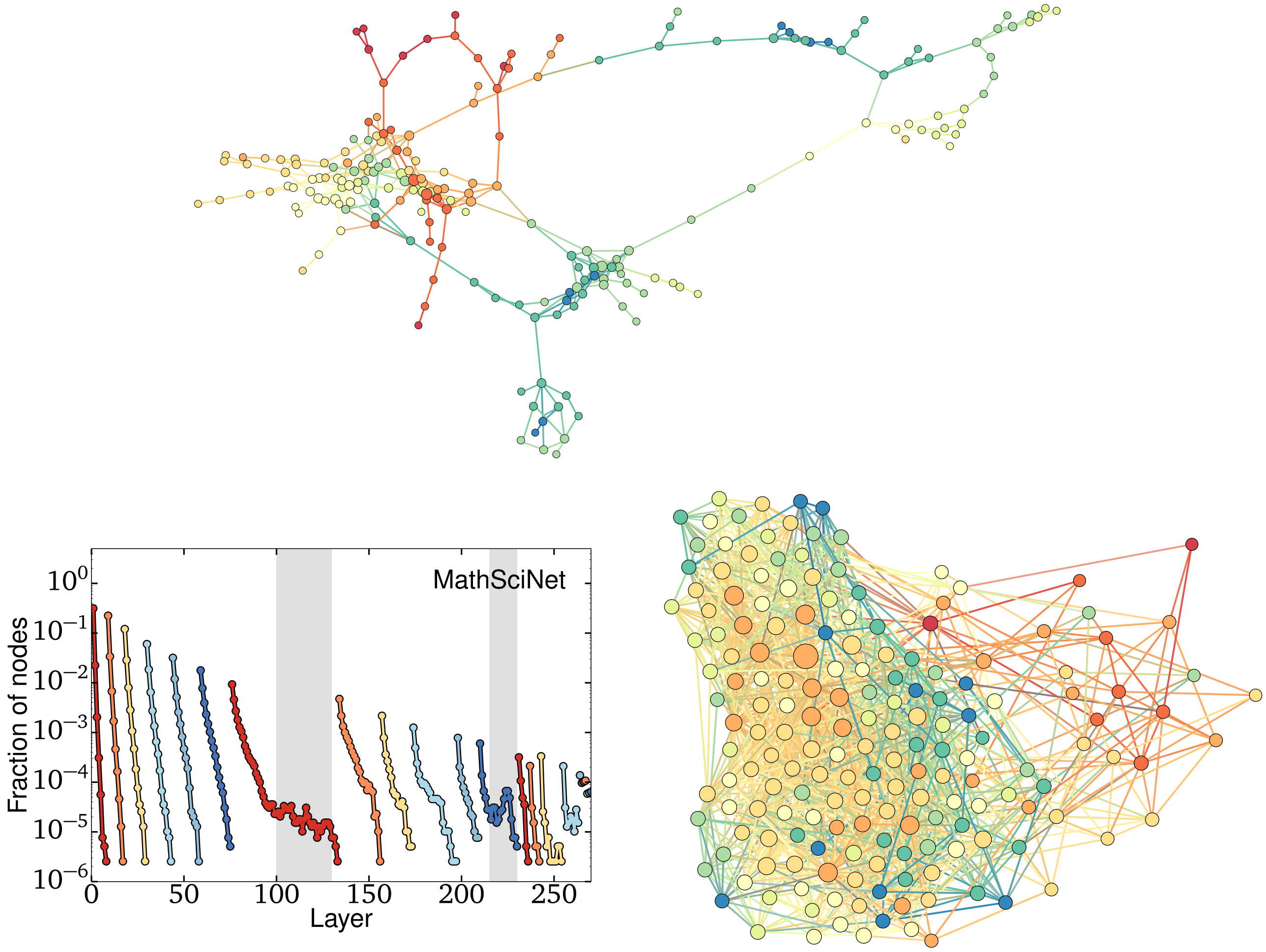

Supplementary Figure 6. **The MathSciNet co-autorship onion spectrum and two selected subgraphs.** While the overall structure of this sparse network is tree-like, some unique subsets can be identified in the spectrum, two of which are shown (shaded layers, the one from the 7-shell is shown on top and the more central community is shown at the bottom right).

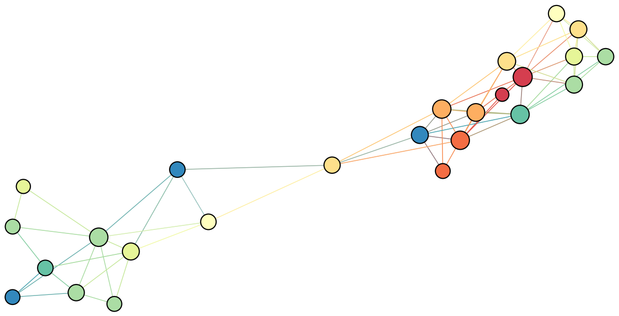

Supplementary Figure 7. **An additional subgraph from layers 125 to 135 of the arXiv.** Similarly to the subgraph shown in the main text, this one corresponds to connected communities of nodes with similar centrality.

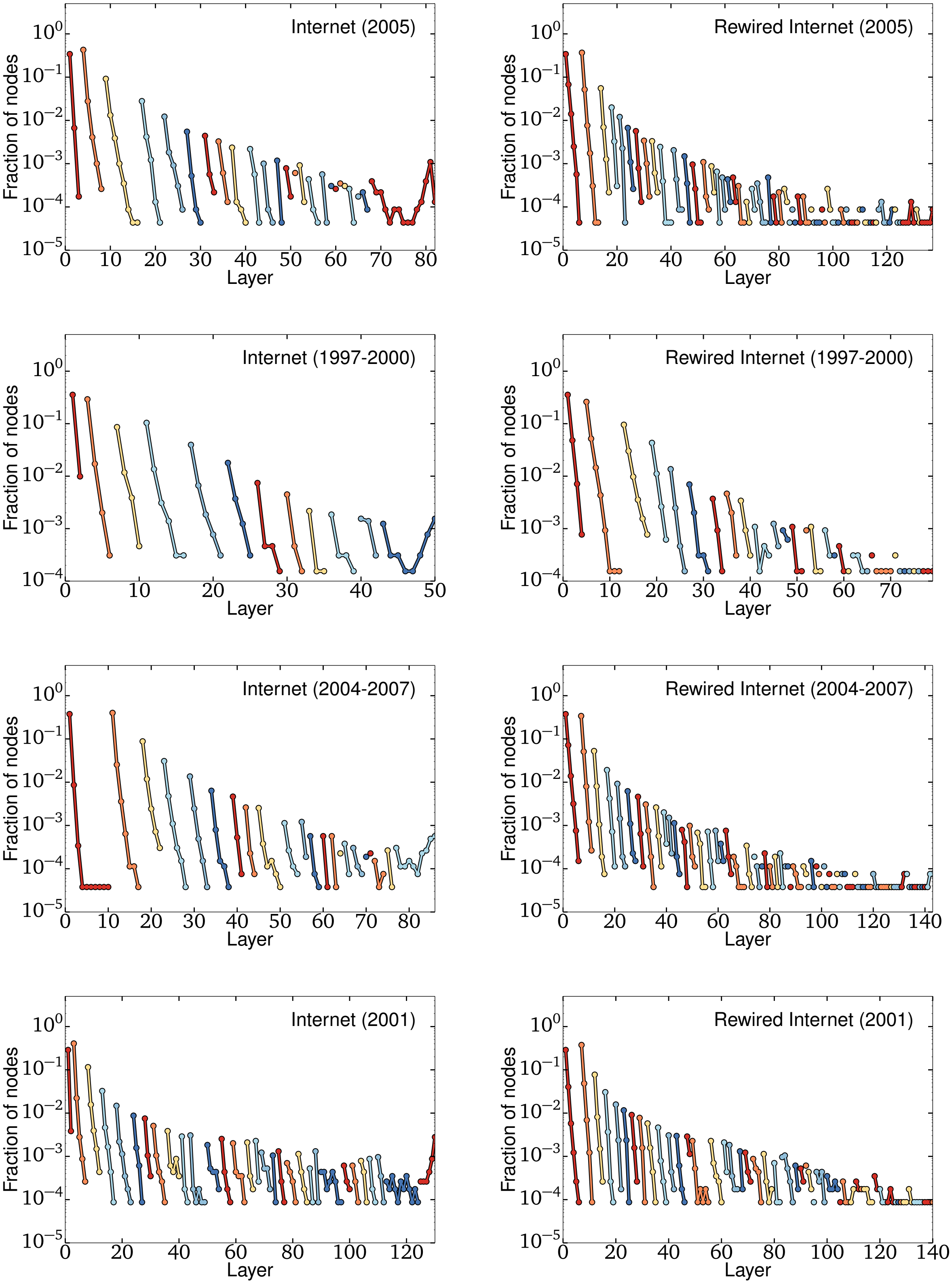

Supplementary Figure 8. **Comparing the onion spectra of snapshots of the Internet structure [5, 6] (left) and their rewired version (right).**

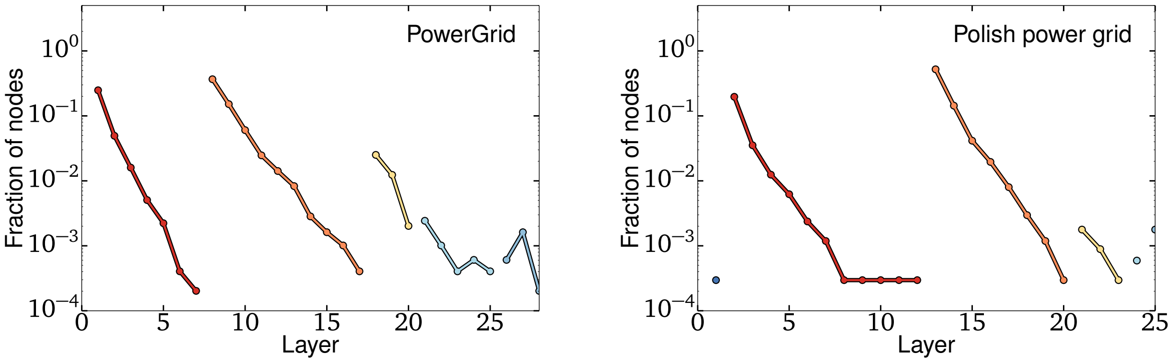

Supplementary Figure 9. **Comparing the onion spectra of two power grids from (left) North Western America and (right) Poland.**

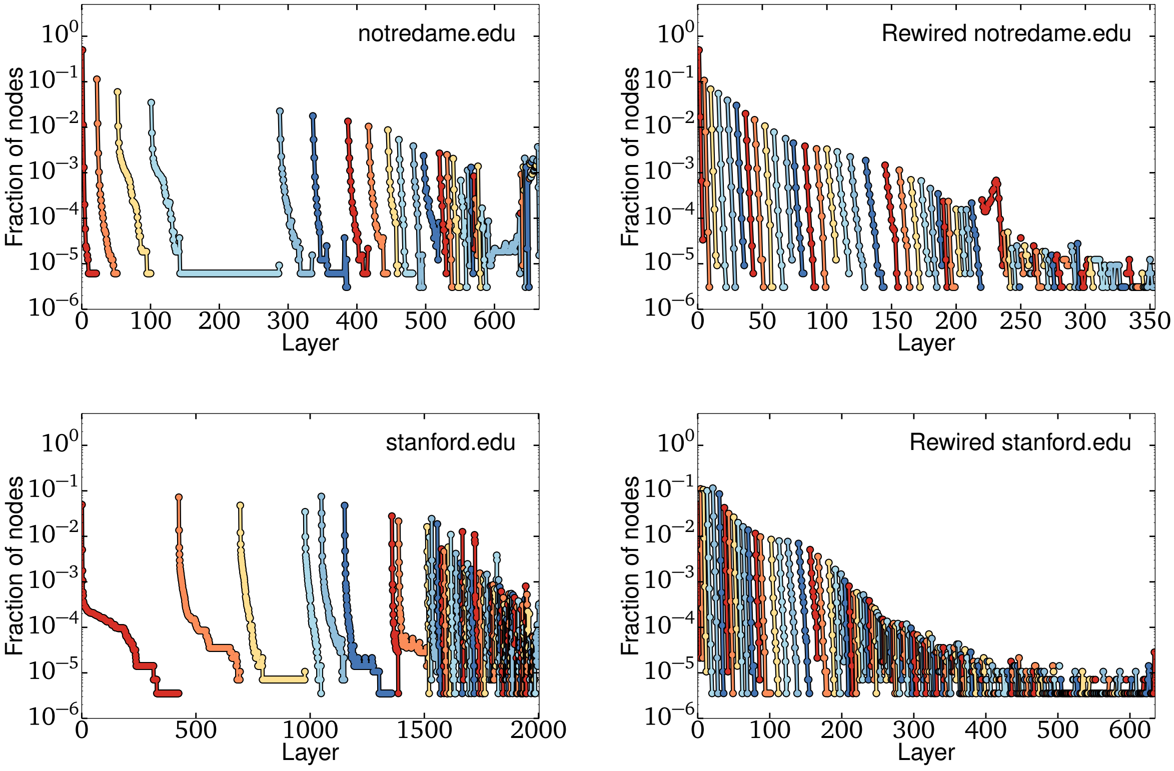

Supplementary Figure 10. **Comparing the onion spectra of domains of the World Wide Web (left) and their rewired version (right).**

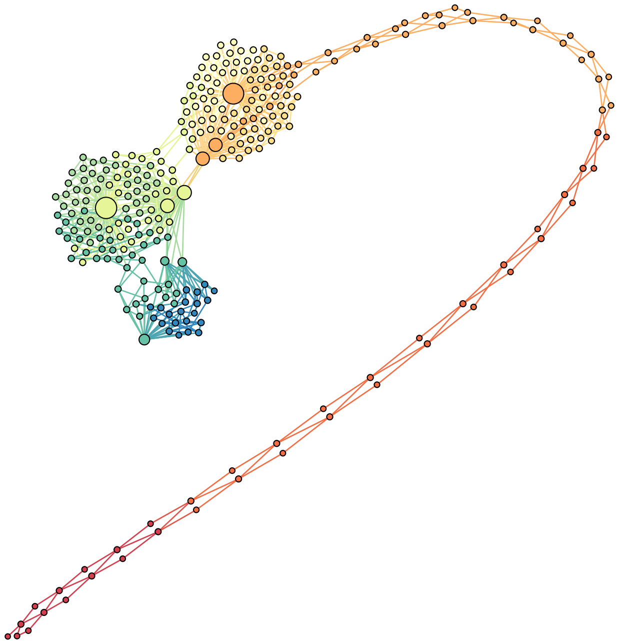

Supplementary Figure 11. **An additional subgraph from layers 1200 to 1300 (in the 6-shell) of `stanford.edu`.** This one corresponds to a mixture of communities coupled to a long chain of central webpages.

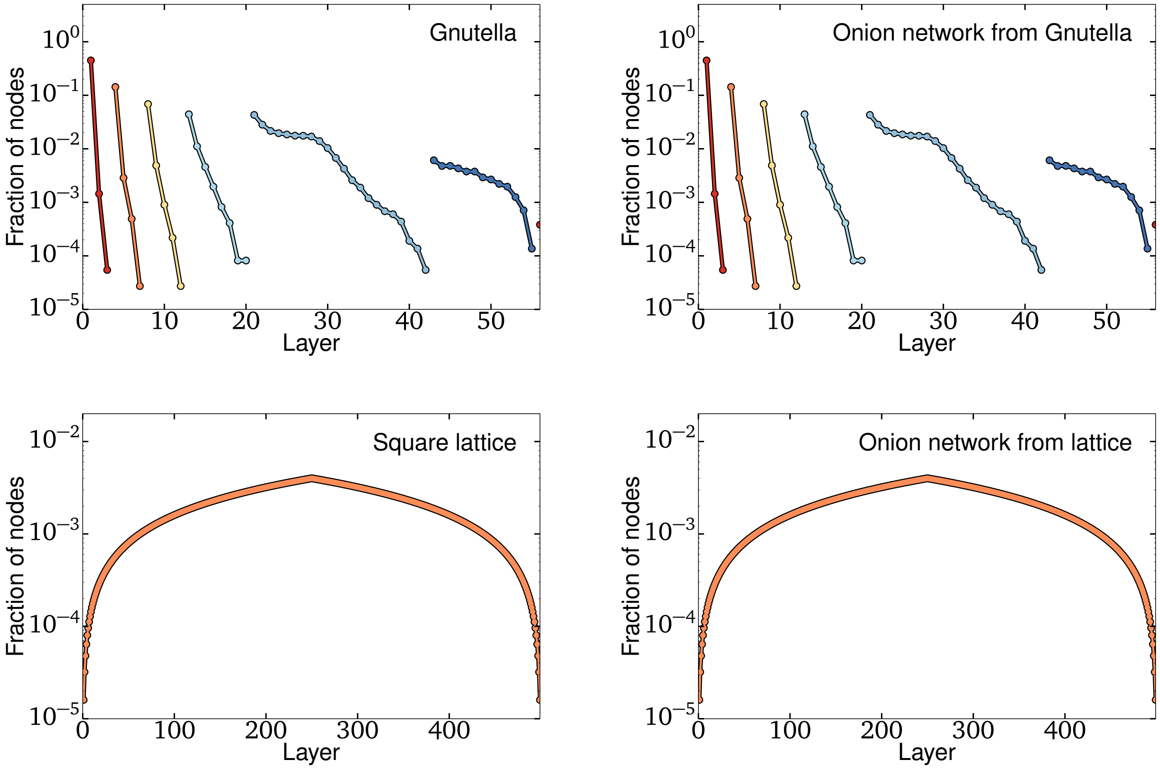

Supplementary Figure 12. **Validating the onion network model (right) with two datasets (left).**

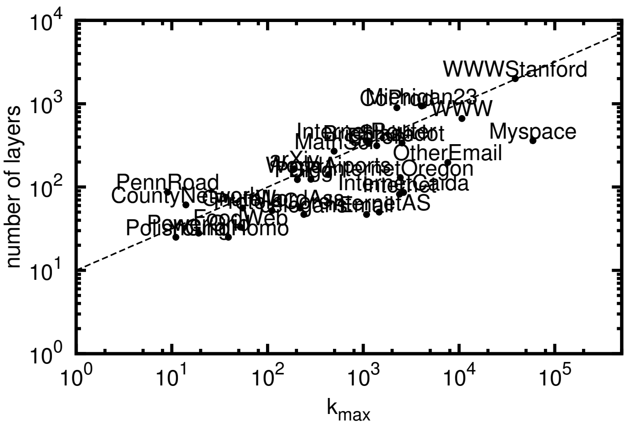

Supplementary Figure 13. **Scaling of the number of layers with maximum degree in all used datasets and more.** Datasets are identified with a small descriptive string, and a conservative scaling behaviour (i.e. $\ell_{\max} \sim \sqrt{k_{\max}}$) is shown with a dotted line.

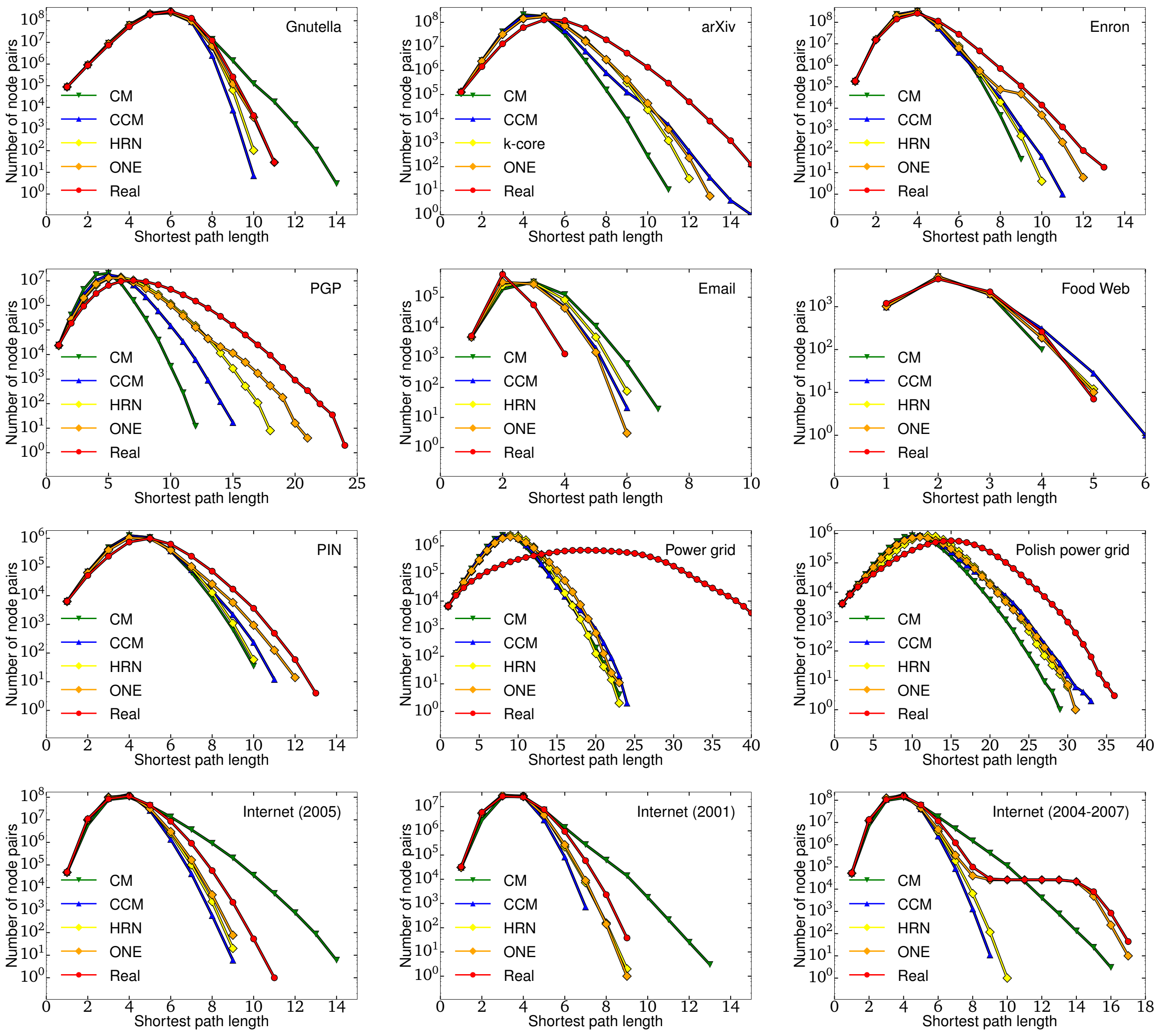

Supplementary Figure 14. **Distribution of shortest path lengths in real complex networks and in two rewired versions.** Real data is shown in red, a network sampled from the ONE is shown in orange and a rewiring conserving degree distribution is shown in blue.
